# Effect of Early Treatment of Spasticity After Stroke on Motor Recovery: Protocol for the Baclotox Multicenter, Double-Blind, Double-Dummy Randomized Controlled Trial

**DOI:** 10.2196/62951

**Published:** 2025-05-09

**Authors:** Emmeline Montane, Nabila Brihmat, Camille Cormier, Claire Thalamas, Vanessa Rousseau, Gerard Tap, Xavier De Boissezon, Evelyne Castel-Lacanal, Baclotox Group, Philippe Marque

**Affiliations:** 1 Department of Physical and Rehabilitation Medicine Toulouse University Hospital Centre Toulouse France; 2 Toulouse NeuroImaging Centre French National Institute of Health and Medical Research (INSERM) Université de Toulouse Toulouse France; 3 Institute for Neurodegenerative Diseases French National Centre for Scientific Research (CNRS) University of Bordeaux Bordeaux France; 4 Department of Physiological Exploration Toulouse University Hospital Centre Toulouse France; 5 Clinical Investigation Centre 1436 Toulouse University Hospital Centre Toulouse France; 6 See Authors' Contributions

**Keywords:** stroke, rehabilitation, muscle spasticity, botulinum toxin A, baclofen, hemiplegia, motor recovery

## Abstract

**Background:**

Individuals with poststroke hemiplegia often develop spasticity, which increases disability. Antispastic treatments such as baclofen and botulinum toxin are commonly prescribed in poststroke recovery. However, their impact on motor recovery, especially when administered within the first 2 months after stroke, remains unclear.

**Objective:**

This study aims to compare the motor recovery effects of botulinum toxin versus oral baclofen. The hypothesis is that botulinum toxin is more supportive of motor recovery than baclofen and enhances functional recovery.

**Methods:**

The study is a multicenter, controlled phase IV, comparative, prospective, randomized, double-blind, double-dummy, superiority trial to compare the toxin and baclofen, and a noninferiority trial to compare the toxin and the placebo. It focuses on the time course of the Fugl-Meyer Motor Assessment (FMA) as the primary outcome. The main inclusion criterion is patients with a single stroke in the past 2 months. Treatment comprises 1 intramuscular injection at treatment initiation and oral tablets for 4 months. Randomized patients are allocated to 3 arms: botulinum toxin with placebo baclofen, baclofen with placebo botulinum toxin, and placebo baclofen with placebo botulinum toxin. FMA scores are assessed at pretreatment, 1 month, and 3 months later. Spasticity, functional abilities, activities of daily living, pain, and quality of life are also evaluated. Adverse effects are monitored. A positive difference of 13 points in the FMA time course between the botulinum toxin and baclofen groups is considered a relevant effect. The data analysis plan involves linear regression models to compare primary and secondary outcomes, with adjustments for covariates such as age, center, and associated treatments. Subgroup analyses will examine proportional recovery profiles, and missing data in Fugl-Meyer scores will be addressed using imputation methods.

**Results:**

A total of 179 participants were randomized across 18 centers, with inclusions delayed due to the COVID-19 pandemic. As of December 2024, the data manager currently has all the data, and a review of data quality is in progress. No statistical analysis has been conducted so far, and the blind will be lifted after the analysis.

**Conclusions:**

This study identifies the most suitable spasticity treatment, considering the specificities of the stroke and constraints during the recovery phase. It will provide recommendations for the primary treatment of early spasticity post stroke.

**Trial Registration:**

ClinicalTrials.gov NCT02462317; https://clinicaltrials.gov/study/NCT02462317; European clinical trials (EudraCT) 2010-022881-28; https://www.clinicaltrialsregister.eu/ctr-search/trial/2010-022881-28/FR

**International Registered Report Identifier (IRRID):**

DERR1-10.2196/62951

## Introduction

### Background

Stroke is the leading cause of acquired motor disability in adults, with 80% of patients experiencing corticospinal tract damage that results in hemiparesis [1,2]. A significant proportion of people who have had a stroke face long-term disability, with many requiring assistance with daily activities, walking, or occupational activities [3]. Early subacute poststroke rehabilitation, which occurs during the period between 7 and 90 days post stroke, focuses on promoting motor recovery and reducing activity limitations, often through intensive rehabilitation and, where appropriate, pharmacological intervention. This phase is considered a critical window for neuroplasticity and motor recovery [4]. While there is substantial evidence from animal models suggesting that certain drugs impact motor recovery, human studies are limited, especially in the context of spasticity management during the subacute phase post stroke [5].

Spastic motor syndrome involves spasticity, dystonia, co-contraction, and clonus due to diminished tonic stretch reflexes and increased muscle tone due to upper motor neuron damage [6]. Although spasticity is typically treated in the chronic phase, there is an increasing trend toward early intervention during the subacute phase [7]. Early management may potentially mitigate long-term functional limitations; however, it remains uncertain whether early pharmacological treatment of spasticity positively or negatively influences motor recovery [8].

Two primary pharmacological treatments for spasticity in France are baclofen and botulinum toxin A, each with distinct mechanisms and evidence of efficacy [9].

Baclofen is a GABA (γ-aminobutyric acid)-B agonist that reduces spasticity by enhancing presynaptic inhibition, thereby decreasing the excitability of afferents in spinal reflexes. Despite its widespread use and endorsement by the French National Health Authority, Haute Autorité de Santé, as a first-line treatment, studies in animal models have raised concerns about the impact of GABAergic drugs on motor recovery. Research indicates that GABAergic agents, such as diazepam, can impair motor recovery and brain plasticity postinjury [10,11]. Baclofen has shown similar detrimental effects on visuomotor learning in healthy human participants, suggesting a potential risk for individuals undergoing motor recovery post stroke [12]. However, the absence of direct studies on baclofen’s effects during the recovery phase post stroke leaves a critical gap in understanding its impact on motor outcomes.

Botulinum toxin A, meanwhile, acts by blocking acetylcholine release at the neuromuscular junction, thereby temporarily paralyzing targeted muscle fibers and reducing spasticity [13]. Its effects generally become noticeable within days and persist for approximately 4 months [14]. Randomized controlled trials demonstrate botulinum toxin’s effectiveness in reducing spasticity in patients with chronic stroke; yet, its effects on motor function are less clear [9,13,15,16]. Most studies have focused on its use in the chronic phase over 6 months post stroke, a period when plasticity decreases and muscular changes may be irreversible. Notably, research using transcranial magnetic stimulation and functional magnetic resonance imaging indicates that botulinum toxin injections may have modulatory effects on the central nervous system, potentially enhancing brain plasticity and supporting recovery [17-21]. This evidence suggests that early use of botulinum toxin in the subacute phase could offer motor recovery benefits by influencing neuroplastic processes.

Given the limited understanding of how these treatments affect motor recovery in the subacute phase, this study aims to investigate the comparative efficacy of baclofen and botulinum toxin A on motor recovery post stroke. Our hypothesis is that botulinum toxin will demonstrate a superior effect on motor recovery compared to baclofen, as it may mitigate spasticity without the potential negative effects on plasticity observed with GABAergic drugs.

### Objectives

#### Main Objective

This study aims to demonstrate the superiority of botulinum toxin A over baclofen on motor recovery in the subacute phase after stroke. The primary end point is the change in the Fugl-Meyer Assessment (FMA) motor score from the start of treatment to 3 months later.

#### Secondary Objectives

Our secondary objectives are as follows:

- To compare the effects of botulinum toxin A and placebo on motor recovery (FMA) over 3 months and establish clinical equivalence.
- To evaluate the impact of treatment on motor recovery (main objective and placebo comparison) based on initial FMA-upper extremity (FMA-UE) scores.
- To assess changes in motor recovery (FMA) during the first month of treatment.
- To compare the effectiveness of the treatments in reducing spasticity symptoms over 1 and 3 months using the Tardieu Scale and Modified Ashworth Scale (MAS).
- To evaluate functional improvement 3 months after treatment using the Rivermead Motor Assessment (RMA), Rankin score, and Barthel score.

#### Exploratory Objectives

- To assess the impact of treatments on pain using a visual analog scale (VAS) at 1 and 3 months.
- To evaluate the quality of life over 3 months using the Reintegration to Normal Life Index (RNLI), a scale validated for patients with vascular hemiplegia.
- To compare reaction times at baseline, 1 month, and 3 months to investigate cognitive effects of treatment.
- To measure patient satisfaction with treatment at 1 and 3 months.

## Methods

### Study Design

**Figure 1:**
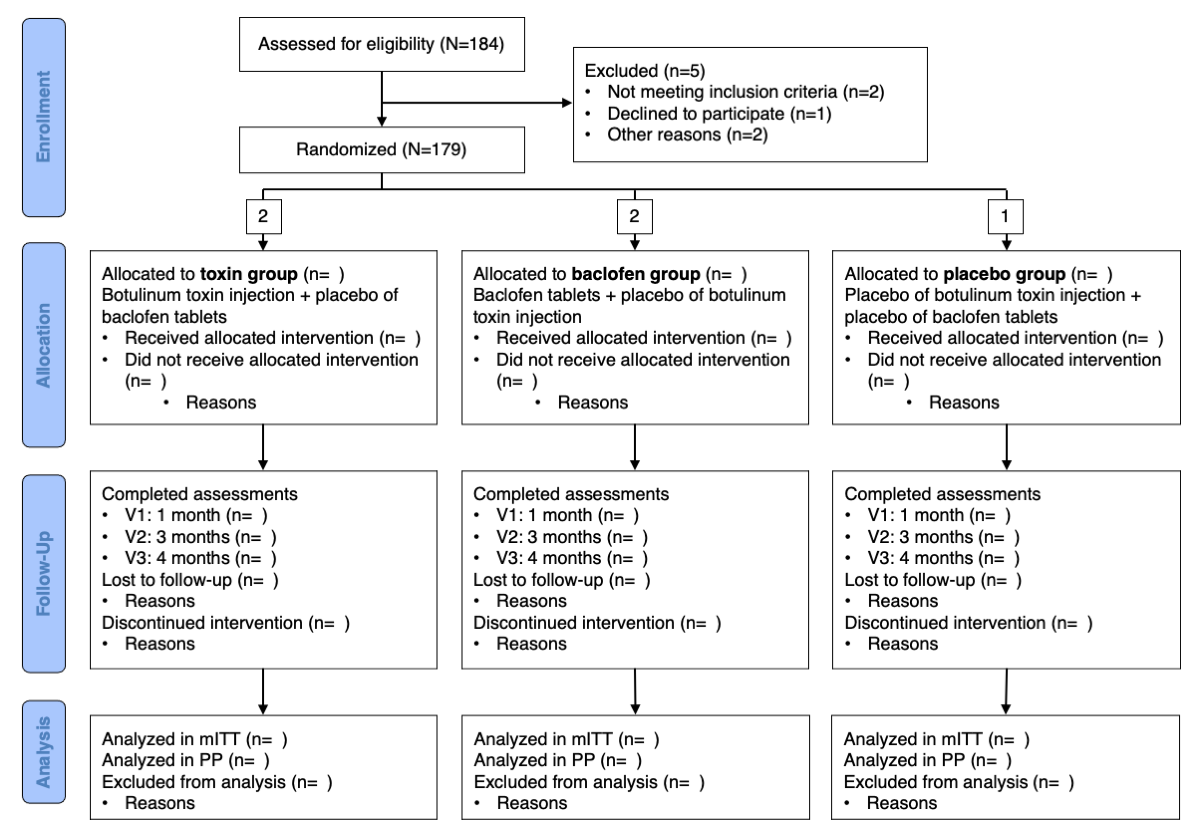
CONSORT (Consolidated Standards of Reporting Trial) flow diagram. mITT: modified intention-to-treat; PP: per-protocol; V1: visit 1; V2: visit 2; V3: visit 3.

This is a multicenter, controlled phase IV, comparative, prospective, randomized, double-blind, double-dummy, superiority trial to compare the toxin and baclofen, and a noninferiority trial to compare the toxin and the placebo. Because of the different modes of administration of these treatments, each group receives both an injection and oral tablets. The study therefore comprises three arms: (1) a “toxin” group treated with an injection of botulinum toxin and placebo oral baclofen; (2) a “baclofen” group treated with oral baclofen and an injection of placebo botulinum toxin; and (3) a double “placebo” group treated with placebo oral baclofen and an injection of placebo botulinum toxin. The CONSORT (Consolidated Standards of Reporting Trials) flowchart in Figure 1 summarizes the enrollment and allocation of the participants.

All patients, regardless of their assigned group, benefit from daily neurorehabilitation care. This care starts simultaneously with the initiation of treatment. For each patient, the protocol duration spans 4 months: 3 months of the controlled protocol and 1 month of gradual discontinuation of the tablets. This gradual discontinuation reflects the standard procedure for stopping baclofen. Participants are recruited from the 18 French physical medicine and neurorehabilitation hospital wards participating in this protocol. The list of study sites can be found in Multimedia Appendix 1.

**Figure 2:**
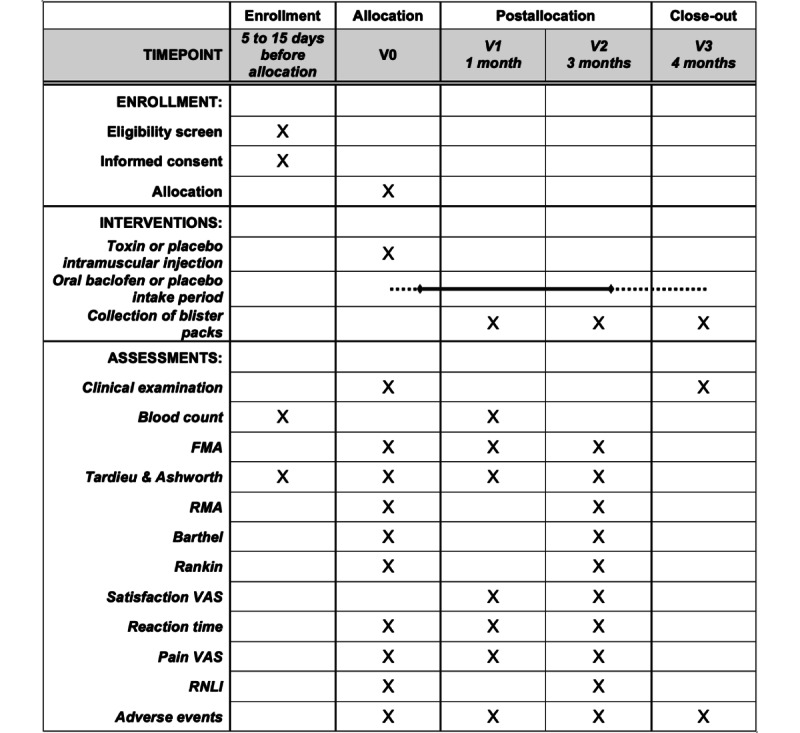
SPIRIT (Standard Protocol Items: Recommendations for Interventional Trials) schedule of enrollment, interventions, and assessments. FMA: Fugl-Meyer Assessment; RMA: Rivermead Motor Assessment; RNLI: Reintegration to normal life; V0: allocation visit; V1: visit 1; V2: visit 2; V3: visit 3; VAS: visual analog scale.

The SPIRIT (Standard Protocol Items: Recommendations for Interventional Trials) guidelines resume the intervention and assessments of the study (Figure 2).

This study is registered in the European EudraCT database (2010-022881-28) and is also registered in ClinicalTrials.gov (NCT02462317).

### Ethical Considerations

This study received Ethics Committee approval (CPP Sud-Ouest et Outre-Mer II) on March 23, 2015 (review number 2-14-33) and authorization from the Agence Nationale de Sécurité du Médicament (ANSM) on December 29, 2014. Conducted in accordance with the Declaration of Helsinki, the study requires both oral and written informed consent from participants. During the preinclusion visit, the investigating physician explains the study’s objectives, procedures, potential risks, benefits, and patient rights. Each participant receives an information sheet and consent form, followed by a minimum 48-hour reflection period to decide on participation. An ancillary study is carried out in the main investigating center. The patient is given a different consent form for this ancillary study and is provided with comprehensive information. In line with the French Public Health Code (articles L.1121-3 and R.5121-13), confidentiality is strictly maintained. Collected data are anonymized, with patient codes generated from initials and inclusion numbers, which are linked only to the center and enrollment order. The sponsor ensures that all participants consented to data access solely for quality assurance. No financial or material compensation was provided; participation was voluntary without incentives.

### Participants

#### Eligibility Criteria

##### Inclusion Criteria

The inclusion criteria are as follows:

- Man or woman between the ages of 18 and 80 years.
- First ischemic or hemorrhagic stroke resulting in hemiplegia.
- Sufficient understanding to participate in the study: Boston Diagnostic Aphasia Examination>3.
- The stroke occurred less than 2 months ago.
- Presence of spasticity warranting treatment: Tardieu score>1(≥2) in one of the following muscle groups: triceps sural, wrist flexors, finger flexors, and elbow flexors.
- The patient’s consent was obtained.
- Affiliated with a social security scheme or equivalent.

The inclusion criteria ensure that participants represent the target population most likely to benefit from the intervention, including adults with recent first strokes and spasticity warranting treatment. These criteria also confirm their capacity to engage meaningfully in the study through sufficient cognitive understanding and access to standard rehabilitation care.

##### Exclusion Criteria

The exclusion criteria are as follows:

- Renal failure at the time of inclusion.
- Currently on antispastic therapy: baclofen, dantrolene, and tizanidine.
- A history of botulinum toxin injection.
- A history of traumatic or vascular brain injury, myasthenia gravis, Lambert Eaton syndrome, or neuromuscular disease.
- Pregnancy
- Surgery with curare within the last month
- Treatment with aminoglycosides, aminoquinolines, or cyclosporine.
- Progressive disease at the time of inclusion.
- A history of convulsive seizure or taking antiseizure medication.
- A history of psychosis or neuroleptic treatment.
- Liver function test abnormalities, at the investigator’s discretion.
- Blood count abnormalities, at the investigator's discretion.
- A history of intolerance or allergic reaction to lactose.
- Glucose or galactose malabsorption syndrome and lactase deficiency.
- A history of anaphylactic reaction.
- Inability to undergo daily rehabilitation for 2 hours.
- Under guardianship, curatorship, safeguard of justice.
- Receiving anticoagulant treatment: heparin with an electric syringe or antivitamin K at an effective dose.

The exclusion criteria are designed to ensure participant safety and the validity of the study by excluding individuals with medical conditions, treatments, or histories that could compromise the intervention’s safety or effectiveness. Participants unable to follow the study protocol are also excluded. These criteria include the standard exclusions for Botulinum toxin treatment and oral baclofen therapy.

### Study Center Inclusion Criteria

Study centers must have experience in spasticity assessment and conducting botulinum toxin A injections. They must admit an adequate number of patients post stroke in the subacute phase for hospitalization every year.

Participants are recruited from among the outpatients and inpatients of the physical and neurorehabilitation medicine ward. Two or more experts determine eligibility according to the inclusion and exclusion criteria.

### Intervention

At the allocation visit (V0), the investigator administered the intramuscular injections and prescribed the tablets. The tablets were taken daily for 3 months until the second postallocation visit (V2). The dosage of tablets was then gradually decreased over 1 month and the effects were monitored during the close-out visit. All participants also received at least 2 hours of neurorehabilitation daily, 5 days per week, led by specialized therapists. This regimen included both physiotherapy and occupational therapy to support comprehensive motor recovery in accordance with the routine practices of the neurorehabilitation centers.

#### Botulinum Toxin Injection Protocol

The choice of muscle groups to be injected depended on the investigator’s clinical examination. The muscle group that validated the patient’s inclusion (Tardieu>1) was systematically injected. Other muscle groups that met the specific criteria (Tardieu score1) were also systematically injected: the triceps surae, flexor digitorum superficialis, flexor digitorum profondus, flexor carpi radialis, flexor carpi ulnaris, and the brachialis (Multimedia Appendix 2). The maximum total dose permitted is 500 units. Additional muscle groups were injected based on the remaining dose of toxin [22].

Injection site localization was tracked using electrostimulation sometimes combined with muscle ultrasound. Depending on the muscles injected, 1 or 2 injection points would be considered at sites with the highest density of motor endplates. The total number of injection points could range between 15 and 20. If requested by the patient, an analgesia protocol with class II analgesics (paracetamol, codeine, and hydroxyzine) was administered 2 hours prior to the procedure, along with inhalation of an equimolar mixture of oxygen and nitrous oxide (Meopa). This was the standard procedure for toxin injections in the participating centers.

#### Baclofen Intake Protocol

Baclofen was provided in a 10 mg capsule and the chosen dose was 60 mg per day in 3 doses commonly used with success in baclofen studies [9]. Treatment was initiated at a dosage of 10 mg in the evening (1 capsule). The dosage was then increased by 1 tablet (10 mg) every 4 days until a dosage of 60 mg per day was reached. If excess sedation occurred during dosage escalation, the clinician reverted to the previous level for an additional 3 days. After 2 unsuccessful attempts to increase the dosage, the last tolerated dosage was maintained throughout the study. Abrupt discontinuation of treatment was not recommended. On completion of the protocol, the baclofen dosage was gradually reduced by 10 mg (1 capsule) every 3 days.

Exiting the protocol occurred either by the patient’s deliberate choice or by necessity, according to the investigator's decision, in the following circumstances.

- Nonadherence to study conditions: poor compliance with the treatment, unauthorized use of other medications.
- Uncontrolled painful spasticity despite the use of class I and II analgesics.
- Occurrence of a serious adverse effect or event.

Premature discharge may be warranted in the event of a clinically significant adverse effect, such as mental confusion, severe sedation, swallowing difficulties necessitating discontinuation of oral feeding and insertion of a nasogastric tube, leuconeutropenia, or drug-induced hepatitis revealed by the laboratory tests (1-month visit). On study discharge, the patient was promptly referred to the investigator for follow-up consultations and ongoing management.

To ensure compliance, drug blister packs were collected, and the remaining capsules were counted.

#### Permitted Care During the Trial

Certain medications could impact motor recovery, particularly GABAergic treatments, which were closely monitored. Patients already on a long-term prescription (over 1 month) of sleeping or anxiolytic treatment might continue this treatment. Discontinuing these patients’ medications had more drawbacks than benefits. Zolpidem and zopiclone, commonly prescribed to patients with poststroke sleep disorders, were permitted during this trial.

Considering the prevalence of poststroke depression and its influence on functional recovery, specific antidepressants are authorized. Antidepressants in the serotonin reuptake inhibitor class were permitted. These medications could potentially facilitate motor recovery irrespective of their antidepressant properties [23]. However, randomization aimed to evenly distribute these treatments across all groups, which minimized the risk of bias.

For secondary stroke prevention, patients were administered treatment according to current recommendations (antiplatelet agents, antihypertensive agents, and cholesterol-lowering agents). Effective-dose anticoagulants (heparin and anti–vitamin-K) were grounds for noninclusion due to the risk of muscle hematoma during injections. However, if necessary, during the protocol postinjection, effective-dose anticoagulants were prescribed without necessitating withdrawal from the study.

It is important to note that all patients received standardized functional neurorehabilitation for 2 hours daily, which included physiotherapy and occupational therapy sessions in line with current practices.

#### Prohibited Care During the Trial

Anxiolytics and other benzodiazepines were prohibited, except zolpidem or zopiclone, which may have been prescribed for the duration of the protocol. Similarly, neuroleptics and tricyclic antidepressants were contraindicated due to their deleterious effects on recovery. Effective-dose anticoagulants at the time of inclusion were a criterion for noninclusion.

### Outcomes

#### Primary Outcome: FMA

The primary end point is the progression of the FMA score from allocation (V0) to the visit three months after allocation (V2) [24].

This is a tool for assessing the motor function of participants with hemiplegia after a stroke. It is also recommended by the European Stroke Organization as a poststroke evaluation outcome [25]. Sensitivity to change is moderate for the overall scale and better for the upper limb subscale after a recovery period. This scale has excellent test-retest reliability and interrater reliability [26]. The floor effect and the ceiling effect are good [27]. This scale is widely used in current practice in France and the study investigators are accustomed to performing it.

#### Secondary outcomes

##### Fugl-Meyer Motor Assessment

Subscores of FMA, specifically FMA-UE and FMA-lower extremity (FMA-LE), from allocation (V0) to the visit 3 months after allocation (V2) are secondary end points.

The progression of the FMA score from allocation (V0) to the visit 1 month after allocation (V1) is a secondary end point.

##### Tardieu Scale

Spasticity is assessed using the Tardieu Scale [28]. This scale is of better metrological quality than the Ashworth scale usually used in this type of study [29]. It allows the analysis of the resistance to stretching at several different speeds. Two speeds are used: XV1 is the slowest possible stretching speed and XV3 is the fastest possible stretching speed. Two results are noted: first, the Tardieu angle, which is the difference between the angle of passive joint amplitude obtained at XV1 and the angle of the first resistance to stretching at XV3, and second, the Tardieu intensity score of the resistance to stretching measured by an ordinal score from 0 to 4.

The end point was the progression from the allocation to 1 month later (V0 to V1) and from the allocation to 3 months later (V0 to V2), focusing on muscle groups such as the triceps surae (knee flexed and extended), carpal flexors, and elbow flexors. This score can be applied to muscles that have not been injected, but given the global action of baclofen, measurement of all these muscle groups was essential.

##### Modified Ashworth Scale

The MAS evaluates muscle tone of the same muscles evaluated with the Tardieu Scale. However, it primarily assesses the intensity of resistance to stretching without considering stretching speed [30]. It is therefore much less precise than the Tardieu Scale in terms of the increase in the myotatic reflex, but it allows a very global consideration of the modifications of the intrinsic biomechanical properties of the muscle fibers and the dystonia of the muscles examined. The end point was the progression from the allocation to 1 month later (V0 to V1) and from the allocation to 3 months later (V0 to V2) on the same muscle groups.

##### RMA

The RMA, validated in individuals with vascular hemiplegia [31], appraises motor performance across various functional aspects, including balance, walking, trunk and lower limb motor skills, and upper limb activities. The end point was the progression from the allocation to 3 months later (V0 to V2).

##### Rankin Score

The Rankin score is a widely accepted scale for the global assessment of poststroke disability [32]. It features 6 degrees and is an evaluation tool recommended by the Haute Autorité de Santé. The end point was the progression from the allocation to 3 months later (V0 to V2).

##### Barthel Score

This scale measures autonomy in the activities of daily living and has been validated in individuals with vascular hemiplegia [33]. It comprised 10 items and demonstrated excellent reproducibility. The end point was the progression from the allocation to 3 months later (V0 to V2).

##### Pain via VAS

Changes in pain were evaluated using a VAS from the allocation to 1 month later (V0 to V1) and from the allocation to 3 months later (V0 to V2).

##### Quality of Life Measurement Via the RNLI Questionnaire

The RNLI questionnaire assessed the quality of life specifically for individuals with vascular hemiplegia [34] and was measured from the allocation to 3 months later (V0 to V2).

##### Reaction Time Measurement

Sound and visual reaction time assessments via laptop aimed to quantify the overall impact of spasticity treatments on cognitive functions. The change in measurements was assessed from the allocation to 1 month later (V0 to V1) and from the allocation to 3 months later (V0 to V2).

##### Treatment Satisfaction Via VAS

Patient satisfaction with spasticity treatment was compared using a VAS at both postallocation visits (V1 and V2).

##### Safety

Adverse events (AE) were systematically monitored at the allocation visit (V0), both postallocation visits (V1 and V2), and the close-out visit (V3). Monitoring covered sedation, somnolence, fatigue, muscular weakness, swallowing difficulties, epileptic seizures, liver function, and blood count.

### Sample Size

The study includes three arms as follows: (1) the “toxin” group, which receives an injection of botulinum toxin and placebo oral baclofen; (2) the “baclofen” group, which receives oral baclofen and an injection of placebo botulinum toxin; and (3) the “placebo” group, which receives placebo oral baclofen and an injection of placebo botulinum toxin. All groups receive standard neurorehabilitation care.

This study is a randomized parallel 2-2-1 type design, which corresponds to the “toxin,” “baclofen,” and “placebo” groups, respectively. The main objective, which is to demonstrate the superiority of “toxin” over “baclofen” on motor function, corresponds to a 2-2 comparison. The secondary objective, which is to demonstrate the noninferiority of “toxin” to “placebo” on motor function, corresponds to a 2-1 comparison. The “placebo” arm is limited due to ethical considerations.

The sample size for the “toxin” and “baclofen” arms was calculated with the 2-sided test at a 5% significance level. This analysis compared the superiority of the toxin over baclofen indicated by the FMA gain, between the allocation visit (V0) and the second postallocation visit (V2). The expected variation in FMA was estimated at 30 points between V0 and V2. [35]. For an 85% power and an FMA difference of 12 points between the “toxin” and “baclofen” groups, 120 patients per group were initially required, accounting for missing data and protocol deviations. This 12-point difference was based on the minimal clinically important difference of FMA, which was approximately 6 points for both the upper [36] and lower extremities [37]. Doubling the minimal clinically important difference for a total FMA score ensured a clinically significant change.

To demonstrate equivalence between the “toxin” and “placebo” groups, a 12-point unilateral zone of equivalence was defined. With an SD of 30 points, 60 patients in the “placebo” arm yielded a power of approximately 80% (77.6%), accounting for 10% missing data.

The original target was 300 patients, but by September 30, 2018, only 140 had been included. Given the time and funding constraints, an amendment increased the target FMA difference to 13 points between V0 and V2. This adjustment, while slightly higher, remains within a clinically meaningful range and is achievable under the study’s operational constraints. Accordingly, 85 participants were required in both the “toxin” and “baclofen” groups, factoring in a 10% data loss. The “placebo” group was reduced to 44 patients to maintain the 2-2-1 randomization ratio. A total of 214 patients were therefore needed for trial inclusion.

### Allocation

Randomization follows a 3-arm, type 2-2-1 (“toxin,” “baclofen,” and “placebo”) design. This process is conducted by Creapharm, the manufacturer and packager of the protocol’s therapeutic batches. Creapharm is an independent company approved by the French drug agency (ANSM). Each center receives therapeutic batches containing 5 treatment packages (2-2-1). Randomization is therefore stratified by center.

A copy of the randomization list is kept by Creapharm and the sponsor’s pharmacy. Randomization is performed before the study starts by assigning an inclusion number in the protocol that aligns with a specific therapeutic batch number. The pharmacy in each center is responsible for accurately assigning the therapeutic batch number associated with the inclusion number. This detail is routinely verified by the principal investigator at each center.

### Blinding

Participants, care providers, investigators, outcome assessors, the data manager, and the biostatistician are blinded to the assignment of the intervention. The blind will be lifted after the statistical analyses**.**

During the research, the investigator and the sponsor can request unblinding if necessary, with the agreement of the coordinating investigator. Unblinding might be necessary in case of a serious, life-threatening AE. Otherwise, unblinding only occurs after the database has been finalized at the end of the research.

In this study, a 2-2-1 randomization scheme is used, resulting in 2 treatment groups and 1 placebo group with differing sample sizes. While blinding is strictly maintained for the 2 treatment groups, the smaller size of the placebo group could potentially reveal its identity during data analysis. To minimize bias and preserve blinding integrity, all data analyses will be conducted on coded datasets, with each group labeled as “group 1,” “group 2,” or “group 3,” without specifying treatment identities. This approach ensures that data handling and interpretation are unbiased until the final analysis is complete. Additionally, the randomization and allocation processes are managed independently by a third-party data manager who has no role in data collection or analysis. These measures aim to uphold objectivity and reduce any influence of group size on the interpretation of results.

### Data Collection and Management

All personnel involved in data collection undergo training before the study begins. The data are routinely collected in the management of patients post stroke.

Participants are informed that there will be additional tests during the close-out visit (V3) following the treatment. Moreover, there is a sincere desire among participants to receive the treatment for an adequate duration. Any discontinuation or deviation from the study protocol is documented along with the reasons for discontinuation.

All the information mandated by the protocol is documented in observation logbooks, with explanations provided for any missing data. Data should be recorded promptly and transcribed neatly into the logbooks. If any erroneous data are identified, it should be clearly crossed out and the correct information entered along with the initials, date, and justification by the investigator or the authorized person who made the correction. These records are maintained in paper observation logbooks with 2 copies generated. One copy is kept by the center investigator, while the second is kept by the research monitor after monitoring. Laboratory and clinical examination results are clearly entered in the clinical records, and scales, which are considered source data, are entered directly in the observation logbooks.

Data are entered on paper by the research technician at each center and electronically at the coordinating center. One copy of the observation logbook triplicate is sent to the coordinating center. The clinical research technician at the coordinating center verifies the accuracy of data and validates the computerized data. Data validation aligns with the data management plan developed by the coordinating investigator and the Methodology and Data Management Centre, which comprises a methodologist, a data manager, and a statistician.

The process of freezing or unfreezing data follows the established procedure at the Methodology and Data Management Centre where raw data are frozen in XML format and as an SAS (version 9.4; SAS Institute) table. Daily backups of the dataset are kept for 4 weeks, followed by monthly tape-recorded archiving.

MéDatAS-CIC is a methodology, data management, and biostatistics center to which the sponsor is affiliated, and which acts as a research support team. The Clinical Investigation Centre is responsible for coordinating the conduct of the study. This service is funded by the Programme Hospitalier de Recherche Clinique (PHRC) grant.

### AE Reporting

The investigator was required to promptly notify the sponsor of any serious AEs (SAEs) or significant developments, occurring at any time from the date of informed consent signing, throughout the patient’s scheduled follow-up, and up to 30 days after follow-up completion if the event was likely related to the research. SAEs are defined as events resulting in death, life-threatening situations, hospitalization or prolongation of existing hospitalization, significant or permanent disability, or interventions to prevent such outcomes. Minor AEs are defined as medical occurrences not meeting SAE criteria but which may still relate to study procedures. The sponsor is then responsible for timely reporting of unexpected SAEs and other new occurrences to the ANSM and the relevant ethics committee.

In cases where participants may have been affected, the ethics committee will ensure that the participants are informed of relevant AEs and confirm their ongoing consent.

### Statistical Methods

#### Statistical Methods for Primary and Secondary Outcomes

The baseline characteristics of the participants are described for the 3 treatment groups and the pairwise standardized differences are used to compare the distribution of baseline covariates between treatment groups.

The variation in the Fugl-Meyer score between the “toxin” and “baclofen” groups is compared using linear regression models. First, the model contains the treatment group as the explanatory variable and the Fugl-Meyer delta score as the response variable. The adjusted model then incorporates the patient’s age, the center, and any associated treatment as explanatory variables, in addition to treatment. The associated treatment is represented by a binary variable indicating the use of at least 1 serotonin reuptake-inhibiting antidepressant (yes or no) or at least 1 benzodiazepine (yes or no). This list is validated during the descriptive analysis of concomitant treatment.

The primary objective tests the superiority of the “toxin” versus “baclofen” in motor recovery at 3 months using 2-sided tests at a 5% α level. The secondary objective aims to demonstrate the clinical equivalence of the “toxin” versus the “placebo” in motor recovery after 3 months, using a 1-sided noninferiority test at a 5% α level and a margin of 13 FMA points to assess noninferiority.

To assess the efficacy of these treatments on spastic symptoms (MAS, Tardieu angle, and Tardieu Scale), 2-tailed *t* tests at a significance level of 5% are conducted for pairwise comparisons. The first analysis is carried out on all muscle groups, and a second analysis is carried out on the muscle groups that have been injected.

For the FMA at 1 month and the variation score between allocation and the second postallocation visit which consists of the RMA the Barthel Index, the Rankin score, the reaction time, satisfaction, pain, and the RNLI, the analyses will be descriptive using the usual statistical measures in each group with a 95% CI.

The modified intention-to-treat population consists of all randomized participants who meet the inclusion criteria, having taken at least 1 dose of the study treatment, and with at least 1 postrandomization evaluation. The per-protocol population includes all patients who complete the study as specified in the protocol, excluding those who do not meet the inclusion criteria, for whom there are major protocol deviations during follow-up, or who do not adhere to the treatment regimen as per protocol. The safety analysis population includes all randomized participants who received at least 1 dose of the study treatment. The modified intention-to-treat population is used to analyze all study objectives. Only the secondary objective, which assesses the noninferiority between the “toxin” and the “placebo is analyzed in the per-protocol population, while AEs are analyzed in the safety population. All statistical analyses are conducted in SAS.

#### Methods for Additional Analyses (eg, Subgroup Analyses)

The proportional recovery rule serves as a predictive model for understanding poststroke recovery. It examines the correlation between initial impairment and subsequent recovery by analyzing FMA-UE both at baseline and in follow-up sessions and defines recovery as the change over time [38]. This research suggests that, on average, a significant subset of patients typically regains approximately 70% of their maximum potential recovery from impairment, while a smaller subgroup experiences notably less recovery. For recovery to be considered proportional, patients must recover roughly 70% of their maximum possible potential at the 3-month mark [38]. Patients who initially show mild to moderate deficits according to the FMA-UE (>10) at baseline often follow a pattern known as “recoverers” [39,40]. Those who display a proportional recovery profile tend to have corticospinal tracts that are less affected throughout the entire course [40,41].

Identifying participants with a proportional recovery profile based on the initial FMA-UE seems to be a promising method for participants with stroke, allowing for a more accurate assessment of treatment effectiveness [42]. This subgroup analysis helps to avoid missing treatment effects within the larger group by focusing on those with a “recoverers” profile.

These groups are determined based on whether the FMA-UE is greater than or equal to 11 or strictly less than 11 during the allocation visit [40].

Within the “recoverers” and “nonrecoverers” groups, linear regression models are used in a comparative analysis between the “toxin” and “baclofen” groups regarding the variation in the FMA-UE score. Initially, an unadjusted analysis uses treatment as the sole explanatory variable and the FMA-UE delta score as the response variable. The adjusted model incorporates the patient’s age, the center, and the presence of associated treatment as explanatory variables, in addition to treatment.

The same unadjusted and adjusted models are also computed using the FMA-LE as the response variable in both the “recoverers and the nonrecoverers” groups.

#### Methods to Handle Missing Data

The primary and secondary outcomes involve examining the difference in a quantitative variable measured at 2 distinct time points. Since the measurements are based on quantitative longitudinal scores, we use the CopyMean approach in the R software (R Foundation for Statistical Computing) [43]. The specific methodology details are determined after assessing the structure of the missing data (such as monotone patterns). Consequently, with this method, scores at the 3 targeted times will be imputed, and subsequently, differences from the initial measurement will be computed. This methodology is only applied to the FMA, the FMA-UE, and the FMA-LE. Other missing data are not imputed.

Details regarding oversight, monitoring, auditing, and protocol amendments are provided in Multimedia Appendix 3.

## Results

The first participant was included on April 20, 2015, and the last on July 9, 2020. A total of 184 poststroke participants were included, with 179 randomized across 18 centers. Regarding the ancillary study, 28 poststroke participants agreed to participate, and 20 healthy participants were included between March 10, 2021, and May 27, 2021.

In December 2024, all data are currently kept by the data manager. The data quality review is underway. It should be completed in January 2025. After this date, the database will be frozen and statistical analyses can begin. The blind will be lifted after statistical analyses.

In December 2024, investigators had no data, no conclusions could be drawn, and no result trends could be identified.

## Discussion

### Principal Findings

This study uses a dual analysis framework: a superiority analysis comparing botulinum toxin with baclofen and a noninferiority analysis comparing botulinum toxin with placebo. This approach provides a comprehensive evaluation of the efficacy of botulinum toxin relative to both an active treatment of spasticity (baclofen) and an inactive comparator (placebo). If mixed outcomes are observed—for instance, if botulinum toxin is found to be superior to baclofen but not to placebo—we will interpret these results separately within the context of each hypothesis. This distinction ensures clarity and rigor in the interpretation of findings and supports transparent discussion of implications for spasticity management.

### Comparison With Prior Work

Previous studies on spasticity treatments after stroke have primarily focused on their effects on muscle tone rather than motor recovery. Botulinum toxin and baclofen are well-established treatments for spasticity, but their impacts on motor recovery remain unclear [9,13,15]. Most randomized controlled trials have investigated botulinum toxin in the chronic phase of stroke recovery (more than 6 months post stroke), where brain plasticity is reduced and muscle changes are often irreversible. Conversely, evidence of baclofen’s effects on motor recovery is largely absent, despite animal studies and small human trials suggesting that activation of GABA receptors may hinder motor learning [10,44,45]. This study uniquely investigates these treatments during the subacute phase, a critical window for neuroplasticity and motor recovery. Spastic symptoms usually begin around 4 weeks post stroke [46,47]. The inclusion criteria of 2 months after stroke provide a balance between including a sufficient number of patients with spasticity and ensuring a sufficient period before the end of the early subacute poststroke phase (more than 3 months) when most of the recovery occurs. By incorporating a placebo group, the study addresses a significant gap in the literature by evaluating the efficacy of these treatments against an untreated baseline. A placebo group was necessary because there is no evidence demonstrating that botulinum toxin or baclofen is superior to placebo for motor recovery during the subacute phase after stroke [48]. Botulinum toxin’s effect on motor recovery compared to placebo is unlikely to show significant differences due to the small sample size and limited study power, though previous literature suggests potential central effects [21]. An ancillary magnetic resonance imaging study aims to investigate the toxin’s impact on brain connectivity, tractography, and activation, making it the first to include both placebo and healthy comparative groups to explore its role in neuroplasticity. The “placebo” group was intentionally kept smaller due to ethical concerns among investigators, who believe it is unethical to withhold spasticity treatment despite the lack of evidence supporting its efficacy for motor recovery. Additionally, a larger placebo group would likely hinder patient recruitment and limit participation from many centers.

### Strengths and Limitations

A major strength of this study is its design, which integrates both placebo and active comparator groups to rigorously assess the effects of botulinum toxin and baclofen on motor recovery. The inclusion of a placebo group—despite ethical and logistical challenges—is crucial for disentangling treatment effects from natural recovery processes. Additionally, the ancillary imaging study offers a unique opportunity to explore the central effects of these treatments on brain plasticity.

However, the study has limitations. While the adjusted sample size is sufficient to detect clinically meaningful differences, it may reduce sensitivity to smaller effects on motor recovery outcomes. Furthermore, the follow-up period of 4 months, though valuable for assessing short-term outcomes, does not capture long-term effects or delayed AEs. Extending follow-up durations in future studies could provide more comprehensive insights into sustained efficacy and safety profiles. Nevertheless, the 3-month follow-up in this study offers valuable early insights into the immediate posttreatment phase, which is a critical period for poststroke motor recovery.

Practical or operational issues arose during the course of the study, reflecting the complexity of its implementation. Securing funding from the PHRC in 2010 marked the first step, but the subsequent year-long process to obtain administrative approvals and transfer funds delayed the study’s initiation. Ensuring blindness between baclofen tablets and their placebo required encapsulating baclofen tablets after a request to affix the manufacturer’s logo was denied. This additional step, involving patient safety measures like verifying expiry dates, added another 2 years. Consequently, the first patient inclusion occurred in 2015. The inclusion rate was slower than anticipated, necessitating a protocol extension and recalculating the required sample size to maintain sufficient statistical power. The COVID-19 pandemic further disrupted the recruitment process in 2020, causing additional delays. Despite these challenges, the study progressed without major technical or procedural issues. Importantly, the question of whether early spasticity treatment affects motor recovery remains unresolved, underscoring the relevance of this research.

### Future Directions

The results of this study will inform recommendations for the timing and selection of spasticity treatments after stroke. Future research should build on these findings by examining the long-term impact of these treatments on functional recovery and quality of life. Larger, multicenter trials with extended follow-up periods are needed to confirm the findings and explore delayed effects. Additionally, further investigation into the neurobiological mechanisms underlying the effects of botulinum toxin and baclofen, particularly through advanced imaging techniques, could deepen our understanding of their roles in poststroke recovery. If botulinum toxin demonstrates its potential to boost natural poststroke recovery in clinical or ancillary studies, it may be possible in a larger protocol to focus on these properties independently of spastic symptoms by including patients earlier after stroke (eg, within 2 weeks).

### Conclusions

This study addresses critical gaps in the understanding of the effects of early spasticity treatments on motor recovery during the subacute phase of stroke. Comparing botulinum toxin to both baclofen and placebo provides robust insights into the efficacy and safety of these treatments. The ancillary magnetic resonance imaging study further deepens our knowledge of the neurobiological mechanisms underlying recovery, offering the potential to guide future therapeutic strategies. Despite challenges in implementation, the study is well-positioned to advance clinical guidelines and improve outcomes for people who have had a stroke.

## Appendix

Multimedia Appendix 1 Study sites and investigators.

Multimedia Appendix 2 Injection sites and recommended dosage.

Multimedia Appendix 3 Oversight and monitoring.

## Abbreviations

AE: adverse event
ANSM: Agence Nationale de Sécurité du Médicament
CONSORT: Consolidated Standards of Reporting Trials
FMA: Fugl-Meyer Assessment
FMA-LE: Fugl-Meyer Assessment-lower extremity
FMA-UE: Fugl-Meyer Assessment-upper extremity
GABA: γ-aminobutyric acid
MAS: modified Ashworth Scale
PHRC: Programme Hospitalier de Recherche Clinique
RMA: Rivermead Motor Assessment
RNLI: Reintegration to Normal Life
SAE: serious adverse event
SPIRIT: Standard Protocol Items: Recommendations for Interventional Trials
V0: allocation visit
V1: visit 1
V2: visit 2
V3: visit 3
VAS: visual analog scale
