# Supplementary material for: Effect of Early Treatment of Spasticity After Stroke on Motor Recovery: Protocol for the Baclotox Multicenter, Double-Blind, Double-Dummy Randomized Controlled Trial
Source: JMIR Res Protoc. 2025 May 9;14:e62951. doi: 10.2196/62951 (PMC12102626; doi:10.2196/62951)
Supplement: Multimedia Appendix 1 [file resprot_v14i1e62951_app1.docx]

**Supplementary material 1: Study sites and investigators**

| **STUDY SITE** | | **LIST OF INVESTIGATORS** |
| --- | --- | --- |
| **N°** | **NAME AND ADRESS** |  |
| **1** | Service de Médecine Physique et de Réadaptation  CHU Rangueil  1 avenue Pr Poulhes 31059 Toulouse | Principal Investigator:  Prof Philippe Marque |
|  |  | Co-Investigator:  Dr Evelyne Castel-Lacanal |
| **2** | Service de MPR  CHU Jean Minjoz  25030 Besançon Cedex | Principal Investigator:  Prof Bernard Parratte |
|  |  | Co-investigators:  Dr Etienne Alenton  Dr Pierre Decavel |
| **3** | Service MPR USN Tastet Girard  Groupe Pellegrin  33076 Bordeaux cedex | Principal Investigator:  Prof Patrick Dehail |
|  |  | Co-Investigators:  Dr Claire Delleci  Dr Helene Cassoudessale  Dr Adrien Petit |
| **4** | Centre de Rééducation MARIENIA  Route de Navarre  64250 Cambo Les Bains | Principal Investigator:  Dr Michel Begue |
| **5** | Institut de Rééducation, Hopital sud, CHU Grenoble  BP 338  38434 Echirolles cedex | Principal Investigator:  Prof Dominic Perennou |
|  |  | Co-Investigator:  Dr Patrick Davoine |
| **6** | Service de MPR – Hôpital R. Poincarré  92380 Garches | Principal Investigator:  Dr Alexis Schnitzler |
| **7** | Service de MPR Hôpital Rothschild  5 rue Santerre  75012 Paris | Principal Investigator:  Prof Gilberte Robain |
|  |  | Co-Investigator:  Dr Hélène Le Liepvre |
| **8** | Service de Rééducation et Convalescence Neurologique Hôpital Swynghedauw  CHU Lille | Principal Investigator:  Dr Etienne Allart |
| **9** | Service de MPR CHU J. Rebeyrol  87042 Limoges | Principal Investigator:  Prof Jean-Christophe Daviet |
|  |  | Co-Investigator:  Dr Romain Joste |
| **10** | Service MPR – CHU Gui de Chauliac  80 avenue Augustin Fliche  34295 Montpellier cedex | Principal Investigator:  Dr Isabelle Laffont |
|  |  | Co-Investigator:  Dr Véronique Carré |
| **11** | Service de MPR - Hôpital Universitaire Carémeau –  Place du Pr. Robert Debré - 30029 Nîmes Cedex 9  CHU de Nîmes  Route de Carnon  30240 Le Grau du Roi | Principal Investigator:  Prof Frédéric Pellas  Co-Investigator:  Dr Claire Jourdan |
|  |  | Principal Investigator:  Dr Huei-Yune Bonnin  Co-Investigator:  Dr Jérôme Froger  Dr Marion Delorme |
| **12** | Service de MPR - GH Lariboisière F.Widal  APHP - Université Paris 7 | Principal Investigator:  Prof Alain Yelnik |
|  |  | Co-Investigator:  Dr Marylène Jousse |
| **13** | Service de MPR - Groupe Hospitalier Pitié-Salpêtrière (APHP)  47 bd de l’Hôpital  75013 Paris | Principal Investigator:  Dr Dominique Mazevet |
|  |  | Co-Investigators:  Dr Kosta Vassilev  Dr Hélène Robert Dr Blaise Bignami |
| **14** | Pôle de MPR, EA 3797, Université Reims Champagne Ardenne,  48 rue de Sébastopol  51092 Reims Cedex | Principal Investigator:  Prof François Boyer |
| **15** | Pôle MPR St Hélier  54 Rue Saint Hélier  35000 Rennes | Principal Investigator:  Dr Philippe Gallien |
|  |  | Co-Investigators:  Dr Tiphaine Berthier  Dr Claire Le Meur  Dr Anne-Laure Roy  Dr Sabine Petrilli  Dr Emilie Leblong |
| **16** | Service de MPR Institut Universitaire de Réadaptation Clemenceau  45 bld Clémenceau  67082 Strasbourg | Principal Investigator:  Dr Stéphane Idée |
| **17** | Service de Réhabilitation Neurologique et Vasculaire Hôpital Léon Bérard  BP 10121  83418 Hyères cedex | Principal Investigator:  Dr Isabelle Thiry-Escudie |
| **18** | Service de Médecine Physique et de Réadaptation  CHU de BREST | Principal Investigator:  Dr Myriam Thiebaut |
