# Supplementary material for: Effect of Early Treatment of Spasticity After Stroke on Motor Recovery: Protocol for the Baclotox Multicenter, Double-Blind, Double-Dummy Randomized Controlled Trial
Source: JMIR Res Protoc. 2025 May 9;14:e62951. doi: 10.2196/62951 (PMC12102626; doi:10.2196/62951)
Supplement: Multimedia Appendix 2 [file resprot_v14i1e62951_app2.docx]

**SUPPLEMENTARY MATERIAL B: INJECTION SITES AND RECOMMENDED DOSAGE**

***Muscle group used to confirm patient inclusion***

| **LOWER LIMB** | Dose to be used | Number of injection sites |
| --- | --- | --- |
| Soleus | 50-100 | 2 |
| Gastrocnemius medialis | 50-100 | 1 to 2 |
| Gastrocnemius lateralis | 50-100 | 1 to 2 |

| **UPPER LIMB** | Dose to be used | Number of injection sites |
| --- | --- | --- |
| Brachialis | 50-100 | 2 |
| Biceps brachialis | 50-100 | 2 to 4 |
| Brachioradialis | 20-50 | 1 to 2 |
| Flexor carpi ulnaris | 20-60 | 2 |
| Flexor carpi radialis | 30-80 | 2 |
| Flexor digitorum superficialis | 30-80 | 2 to 3 |
| Flexor digitorum profundus | 20-60 | 2 |

***Other muscle groups***

| **LOWER LIMB** | Dose to be used | Number of injection sites |
| --- | --- | --- |
| Rectus femoris | 100-200 | 2 to 4 |
| Tibialis posterior | 50-150 | 2 |
| Flexor digitorum longus | 50-100 | 2 |
| Flexor digitorum brevis | 50-100 | 1 to 2 |

| **UPPER LIMB** | Dose to be used | Number of injection sites |
| --- | --- | --- |
| Pectoralis | 100-200 | 2 to 3 |
| Pronator teres | 20-60 | 1 to 2 |
| Pronator quadratus | 20-50 | 1 |
| Interossei | 5 – 10 x4 | 4 |
