# Supplementary material for: Effect of Early Treatment of Spasticity After Stroke on Motor Recovery: Protocol for the Baclotox Multicenter, Double-Blind, Double-Dummy Randomized Controlled Trial
Source: JMIR Res Protoc. 2025 May 9;14:e62951. doi: 10.2196/62951 (PMC12102626; doi:10.2196/62951)
Supplement: Multimedia Appendix 3 [file resprot_v14i1e62951_app3.docx]

# Multimedia Appendix 3

## Oversight and monitoring

### Composition of the coordinating centre and trial steering committee

A monitoring committee has been established, comprising 5 principal investigators, the clinical research technician, the clinical research assistant from the protocol, and 2 individuals with expertise in toxin and motor recovery but not directly involved in the protocol. The monitoring committee meets twice a year to:

- monitor the progress of inclusions per centre

- ensure the safety of enrolled patients after reviewing adverse events (AEs)

- make decisions regarding the study's continuation

Additionally, the protocol monitoring committee can be called upon by the sponsor at any time, especially in cases of a significant number of serious adverse events.

### Composition of the data monitoring committee, its role and reporting structure

MéDatAS-CIC is a structure specialised in the management of research databases independent of the principal investigator. It is funded by the PHRC grant.

The research is overseen by a clinical research technician who, in collaboration with the coordinating investigator, undertakes the following responsibilities:

- research logistics and monitoring

- compiling progress reports

- ensuring the accuracy and completeness of the observation logbook (requesting additional information, making corrections, etc.)

- notifying the sponsor of significant adverse events

The technician adheres to standard operating procedures and collaborates with the clinical research assistant delegated by the sponsor.

### Adverse event reporting and harms

The investigator is required to promptly notify the sponsor of any serious adverse events or developments, regardless of when they occur:

- from the date of signing of the consent

- during the entire duration of the patient's follow-up scheduled by the research

- up to 30 days after the end of the participant’s follow-up scheduled by the research when it is likely to be due to the research

The sponsor is responsible for timely reporting of unexpected adverse events and any new occurrences during the study to:

-the ANSM (French National Agency for Medicines and Health Products Safety)

-the competent Ethics Committee

If necessary, the committee ensures that participating subjects have been informed about adverse events and confirms their consent.

### Frequency and plans for auditing trial conduct

An audit can be carried out at any time by individuals mandated by the sponsor independent of those responsible for the research (such as the PHRC grant organisation). The purpose of an audit is to ensure the quality of the research, the validity of results, and compliance with applicable laws and regulations.

Investigators commit to adhering to the requirements of the sponsor and the competent authority concerning audits or inspections of the research. Audits may encompass all research stages, starting from protocol development through result publication and the categorisation of data utilised or produced in the research.

### Plans for communicating important protocol amendments to relevant parties (e.g. trial participants, ethical committees)

Any substantial modification, i.e. any change likely to have a significant impact on the protection of individuals, the conditions of validity, the results of the research, the quality and safety of the products tested, the interpretation of scientific documents supporting the conduct of the research, or the methods of conducting the research, is the subject of a written amendment submitted to the sponsor. The sponsor must obtain approval from the ethics committee and authorisation from ANSM prior to its implementation.

The ethics committee is notified of non-substantial modifications, which are those having no significant impact on any aspect of the research, for informational purposes.

All amendments to the protocol must be brought to the attention of all investigators participating in the research. The investigators undertake to respect the content of these amendments.

Any amendment that modifies patient management or the benefits, risks, or constraints of the research is the subject of a new information note and a new consent form, which are obtained according to the same procedure as described above.

In France, an independent ethics committee is chosen at random from all the French ethics committees to evaluate the research protocol. The ethics committee that is chosen is independent of the sponsor~~,~~ and cannot be from the same region.
